# A Pilot Randomized Cross-Over Trial to Examine the Effect of Kiwifruit on Satiety and Measures of Gastric Comfort in Healthy Adult Males

**DOI:** 10.3390/nu9070639

**Published:** 2017-06-22

**Authors:** Alison Wallace, Sarah Eady, Lynley Drummond, Duncan Hedderley, Juliet Ansell, Richard Gearry

**Affiliations:** 1 The New Zealand Institute for Plant & Food Research Limited, Lincoln 7608, New Zealand; sarah.eady@plantandfood.co.nz; 2 Drummond Food Science Advisory Limited, Christchurch 7682, New Zealand; lynley_dfsa@me.com; 3 The New Zealand Institute for Plant & Food Research Limited, Palmerston North 4474, New Zealand; duncan.hedderley@plantandfood.co.nz; 4 Zespri International Limited, Mount Manganui 3149, New Zealand; juliet.ansell@zespri.com; 5 Department of Medicine, University of Otago, Christchurch 8140, New Zealand; Richard.Gearry@cdhb.health.nz

**Keywords:** kiwifruit, protein, SmartPill™, gastrointestinal discomfort, satiety

## Abstract

‘Hayward’ kiwifruit anecdotally are associated with improved gastrointestinal comfort following the consumption of high protein meals, possibly because of the presence of a protease enzyme, actinidin. The study aimed to use SmartPill™ technology to investigate the acute effect of kiwifruit with actinidin (*Actinidia chinensis* var. *deliciosa* ‘Hayward’) and kiwifruit without actinidin (*A. chinensis* var. *chinensis* ‘Hort16A’) on digestion of a large protein meal. Ten healthy male subjects were recruited. The participants attended the clinic three times, having fasted overnight. They consumed a test meal consisting of 400 g lean steak and two ‘Hort16A’ or two ‘Hayward kiwifruit’. Subjects completed visual analogue scales (VAS) by rating feelings of hunger, satisfaction, fullness, and comfort and swallowed a SmartPill™ before completing further VAS scales. After 5 h, participants consumed an ad libitum lunch to assess satiety. SmartPill™ transponders were worn for five days. There were no significant differences in gastric emptying time, small bowel, or colonic transit time between the two kiwifruit arms of the study measured by SmartPill™. Similarly, no significant differences were observed in VAS satiety measures or energy consumption at the ad libitum meal. However, the measurement of overall gastric comfort tended to be lower, and bloating was significantly reduced following the consumption of the steak meal with ‘Hayward’ kiwifruit (*p* < 0.028). Conclusions: The SmartPill™ is marketed as a diagnostic tool for patients presenting with gastrointestinal disorders and is usually used with a standard ‘SmartBar’. This small pilot study suggests that it is less likely to measure gastric emptying effectively following a high protein meal, as it may be delayed because of the meal’s physical consistency. However, green kiwifruit, containing actinidin, may reduce bloating and other measures of gastric discomfort in healthy males. Possible future studies could use repeated measures with more readily digested protein and larger numbers of participants.

## 1. Introduction

Gastrointestinal discomfort is a common condition throughout the world, which often leads to a reduction in quality of life. Gastrointestinal discomfort is a target for potential health claims for foods identified by the European Food Safety Authority (EFSA). *Actinidia chinensis* var. *deliciosa* ‘Hayward’ (marketed as Zespri^®^ Green Kiwifruit) is recognised as a fruit that assists with the potential relief of gastrointestinal symptoms [1]. Gastrointestinal discomfort is generally defined as being associated with episodes of abdominal pain or discomfort (such as bloating, abdominal pain/cramp, and borborygmi (rumbling)) in the absence of organic diseases or biochemical abnormalities [2,3]. It is commonly associated with food or drug intake or with alterations of bowel habit and varies among individuals in both frequency and severity. Kiwifruit contains a natural protease (actinidin) which is active in ‘Hayward’ but either inactive or not present in *A. chinensis* var. *chinensis* ‘Hort16A’ (marketed as Zespri^®^ Gold Kiwifruit). Actinidin has been shown in in vitro and animal studies to remain active under gastric and ileal conditions [4] and to influence gastric emptying rates [5].

‘Hayward’ kiwifruit are often used as a natural food to improve bowel function and anecdotally are associated with improved gastric comfort following the consumption of high protein (meat) meals. Although a number of studies have investigated the effects of kiwifruit on bowel habit, no studies have looked at the acute effects of kiwifruit immediately following meat consumption. Animal studies have observed increased rates of gastric emptying in the presence of actinidin-active kiwifruit [5].

Green Kiwifruit (Actinidida delisciosa ‘Hayward’) has been reported to help with the digestion of food and relief of gastric discomfort for many years, and this is thought to be due to the presence of the proteolytic enzyme Actinidin within the fruit. Actinidin has been shown to enhance the digestion of protein during the gastric and small intestinal phases of the digestive process, giving rise to a number of different peptides [6]. Studies have demonstrated the positive effects of actinidin both in vitro and in vivo. Using simulated gastric conditions, Kaur et al. [4] showed that a kiwifruit extract (actinidin) added to protein sources derived from soy, meat, milk, and cereals increased protein digestion by as much as 48%. In vivo studies using rats and pigs have also demonstrated the effect of actinidin on protein digestion. Rutherford et al. [6] examined the effect of freeze-dried kiwifruit extracts from ‘Hayward’ kiwifruit (high actinidin activity) and ‘Hort16A’ kiwifruit (low actinidin activity) on several protein sources (whey protein isolate, beef muscle, gelatine, soy protein isolate, gluten, and zein) when formulated into experimental diets and fed to rats for 13 days. The high actinidin diets increased the gastric digestibility of beef muscle protein, gelatine, soy protein isolate, and gluten by 40%, 60%, 27%, and 29%, respectively, but demonstrated no effect on ileal protein digestibility. Similarly, Montoya et al. [5] showed that actinidin extracts from ‘Hayward’ kiwifruit fed to rats enhanced gastric digestion of beef muscle protein, gluten, and soy protein isolate in addition to enhancing gastric emptying rates for beef muscle protein and zein. Studies in pigs have also corroborated these findings, which may be useful in finding methods to reduce the feelings of over fullness and gastric discomfort reported by consumers of high protein diets [7]. In addition, because green kiwifruit is thought to aid in digestion, it may also increase feelings of satiety after a high protein meal. Another application of these observations would be the enhancement of protein digestion and absorption in human populations with enhanced protein requirements such as elite athletes or in the elderly and the chronically ill who are unable to consume or digest and absorb sufficient protein to meet their dietary requirements. It has been suggested that a moderate 113 g serving of an intact protein (i.e., lean beef) contains sufficient amino acids to increase mixed muscle protein synthesis by approximately 50% in both young and elderly individuals [8]. Hence the addition of ‘Hayward’ kiwifruit to a protein rich meal may increase protein absorption and muscle protein synthesis. 

Using an acute randomized cross-over clinical intervention study, the present trial examined the potential of ‘Hayward’ kiwifruit to aid digestion following a high protein meal in ten healthy adult males. The study aimed to investigate the effects of actinidin, the proteolytic enzyme present in ‘Hayward’ kiwifruit, on gastric emptying times and subjective feelings of gastric comfort and satiety. Gastric emptying, pH and pressure were measured using SmartPill™ technology; satiety and gastric comfort were rated on visual analogue scales; and subsequent energy consumption was measured following an ad libitum meal. We hypothesised that the presence of actinidin in ‘Hayward’ kiwifruit would reduce gastric emptying times and improve feelings of gastric comfort and satiety compared with a control of ‘Hort16A’ kiwifruit, which contains little or no actinidin.

## 2. Materials and Methods

Ten healthy males took part in this study. Participants were recruited through local advertising and through Plant and Food Research participant databases of previous trial participants. Participants were screened for eligibility for the study. Anthropometric measures (height, weight, body mass index) were collected as well as one venous blood sample (approximately 10 mL), which were analysed for a comprehensive metabolic panel (Chem 20 panel) to assess the overall status of the participant’s metabolism. To be eligible, participants had to be aged 18–50 years, have a Body Mass Index (BMI) between 19–30 kg/m^2^, and have normal glucose tolerance, as determined by a fasting glucose below 5.7 mmol/L. Individuals were excluded if they had a major illness like cardiovascular disease, diabetes, cancer, renal failure, previous gastrointestinal surgery (not including appendectomy), and neurological conditions (stroke, multiple sclerosis, spinal cord injury). They were also excluded if they had gastrointestinal disorders (Crohn’s disease; diverticulitis, irritable bowel syndrome) and/or the presence of any alarm features associated with bowel habit (recent changes in bowel habit in <3 months; rectal bleeding, weight loss, haemorrhoids). Individuals also had not to have diabetes or gastroparesis, major food allergies/intolerance, a known allergy to kiwifruit, or extreme dietary habits.

All participants gave their informed consent for inclusion before they participated in the study. The study was conducted in accordance with the Declaration of Helsinki, and the study was approved by the New Zealand Human Disability and Ethics Committee, approval number 14/STH/158 and was registered with the Australia New Zealand Clinical Trials Registry (ACTRN12614001080617).

The study design was a randomized controlled crossover design, with participants consuming a single test meal (steak) with the active ‘Hayward’ kiwifruit and two control ‘Hort16A’ kiwifruit to assess for individual variability. There was a period of at least seven days between each meal. Each of the participants was asked to consume a standard meal on the evening before the test. The content of the meal is shown in Table 1.

At each of the three visits, the participants were asked to arrive at the clinic after fasting from 22:00 h the previous evening, including liquids. At the clinic, participants were asked to complete baseline visual analogue scales (VAS), rating their subjective feelings of hunger, fullness, satisfaction, and prospective food consumption [9]. They were also asked to complete a questionnaire relating to feelings of comfort and bloating [10]. The test meal was then served along with the kiwifruit and 250 mL water, and the participants were asked to consume the meal in full but at their own pace within a 15-min time period. Immediately following the meal, the participants were asked to swallow the activated SmartPill™ and place the transponder upon their body, where it had to remain for the following three days or until visible passing of the SmartPill™ was observed. The participants were also asked to complete the VAS scales and comfort scales at this time (T = 0). These questionnaires were then repeated every hour for the next 5 h. 

The participants were asked to stay at the clinic and were allowed to read or write or talk but they were not allowed to sleep. During this time participants were reminded to log all relevant events on the SmartPill™ transponder. These included:

▪ Eating a meal
▪ Having a bowel motion
▪ Passing gas
▪ Nausea
▪ Cramping, pain
▪ Getting up in the morning
▪ Going to bed at night
▪ Vigorous activity.

No further foods were allowed throughout the morning until the ad libitum lunch was served 300 min later to assess energy and macronutrient intake. Participants were, however, allowed to drink water throughout the morning. Lunch was served individually in a quiet room with minimal distraction. 

Following lunch, participants were free to return to their everyday activity for the following three days but they had to keep the SmartPill™ transponder on their body at all times (except for showering and sleeping, where it had to remain as close as possible to them) and they were asked to log activity as previously listed. On the third day, participants were asked to revisit the clinic to return the transponder for downloading of the data.

Each participant completed this procedure three times until they had completed the standard test meal and two baseline measures. The order in which they received the kiwifruit was randomised to ensure the integrity of the study.

The kiwifruit used in this study were:

▪ *A. chinensis* var. *deliciosa* ‘Hayward’ (marketed as Zespri^®^ Green Kiwifruit)
▪ *A. chinensis* var. *chinensis* ‘Hort16A’ (marketed as Zespri^®^ Gold Kiwifruit).

All fruit were ripened to a ready-to-eat (RTE) stage as determined by pressure testing (0.8–1.0 kgf)/cm^2^ and fruit from each test period were analysed for actinidin activity using the azocasein method [11].

The test meal for this study consisted of 400 g lean rump beef steak (supplied from a single source and cubed into small uniform dice roughly 1.5 cm). The beef was weighed accurately into 400 g portions and then stir-fried for 3 min to brown before being braised for a further 4 min with 60 mL beef stock. Each meat portion was seasoned with ¼ teaspoon of salt and ¼ teaspoon of ground black pepper. All meat meals were pre-prepared on the same day before the start of the study and frozen in individual portions in a domestic freezer. Meals were defrosted overnight and re-heated in a microwave for 2 min before serving.

The standard test meal consisted of the meat meal accompanied by 200 g of ‘Hayward’ kiwifruit flesh and 250 mL water.

The control (baseline) meal consisted of the meat meal accompanied by 200 g ‘Hort16A’ kiwifruit flesh and 250 mL water.

The ad libitum lunch consisted of a buffet style meal with one hot item and several cold choices. Participants were advised that they could eat as much or as little as they chose but that they were to remain at the clinic for a further 30 min and that they were not allowed to remove any remaining food from the room. The items presented at the meal are shown in Table 2. All the food items were served in excess. Prior to and following the lunch, covert weighing of all food items was carried out to allow the calculation of energy and macronutrient intake. The energy and macronutrient content of the foods consumed were calculated using the dietary programme Foodworks version 7.0: (Xyris Software, Spring Hill, Australia).

The primary study outcomes were changes in gastric emptying rate, gastric pH, temperature, and small bowel transit times as assessed by SmartPill™ technology. SmartPill™ is a wireless motility capsule used in the medical community as a diagnostic technology for evaluating gastrointestinal disorders such as gastroparesis and constipation [12]. The SmartPill™ is a cylindrical capsule, which measures pH, pressure, and temperature in real time; the data are wirelessly transmitted to a data receiver that is attached to the participant’s clothing. MotiliGI software (SmartPill, Inc., Buffalo, NY, USA) uses changes in pH and temperature to determine Gastric Emptying time (GET), Small Bowel Transit Time (SBTT), Colonic Transit Time (CTT), and Whole Gut Transit Time (WGTT). GET is defined as the time between capsule ingestion and an abrupt rise in pH above gastric baseline pH. This rise in pH corresponds with the transition from the acidic stomach to the alkaline duodenum. SBTT is the time between duodenum entry and caecum entry. Caecal entry is defined as the first sustained drop in pH of more than 1 unit that occurs at least 30 min after entry into the small bowel. CTT is the difference between entry into the caecum and exit from the body, which is indicated by an abrupt decrease in temperature. WGTT is the time between the ingestion of the SmartPill™ and its exit from the body [13].

The secondary outcomes were changes in subjective feelings of satiety (hunger, fullness, satiation, desire to eat, and amount of food) assessed by visual analogue scales, and subjective feelings of gastric comfort (abdominal pain, rumbling, bloating, belching, flatulence) assessed by a short questionnaire. Participants were asked to rate five questions from ‘not at all’ to ‘extremely’: How hungry are you? How full are you? How satiated are you? How strong is your desire to eat? and How much do you think you could eat right now? A line scale was used with each point equalling 1 centimeter. These visual analogue scales were used to assess subjective feelings of hunger and satiety over the course of the 6-h clinic visit: arrival, immediately after eating, and 60, 120, 180, 240, and 300 min after eating. The results for each individual were recorded and the areas under the curve (AUC) for each participant-meal combination were calculated.

### Statistical Analysis

This study was carried out as a pilot study. Areas under the curve were calculated using the linear trapezoidal rule, using the hourly Visual Analogue Scales (VAS) ratings (to 5 h after eating). The ‘on arrival’ VAS rating was used as a covariate in the analysis of variance. A similar process was followed with the discomfort scores. Data were analysed using analysis of variance (ANOVA), with Participant and Order (first, second, or third meal) as blocking terms/random effects and ‘Hayward’ v. ‘Hort16A’ as the treatment factor. The residuals were inspected to ensure the assumptions of the ANOVA were valid. The least significant differences (LSDs) between the ‘Hayward’ and ‘Hort16A’ means were calculated at the 5% level, as were the 90% confidence intervals for the difference between the ‘Hayward’ and ‘Hort16A’ means. The 90% confidence interval is sometimes used in bioequivalence testing, where the criterion is that both ends of the interval are less than a certain percentage (often 20% for veterinary drugs) of the control mean. The aim is to show that the two treatments have similar means, rather than just means that are not significantly different because of high variability in the data. The differences between the first and second ‘Hort16A’ kiwifruit meal were tested by adding a factor for this nested within the ‘Hayward’ v. ‘Hort16A’ factor. The analysis was done in GenStat (version 16, 2013, VSNi Ltd., Hemel Hempstead, UK).

## 3. Results

The average age of the participants was 34 years (range 21–48 years), and the average body mass index was 24 kg/m^2^ (21–29 kg/m^2^). Table 3 shows the baseline demographic of the participants. All 10 participants completed the study with 100% compliance.

Two batches of the kiwifruit used in the study were analysed for actinidin content during the trial period. One batch was tested in November 2014 and one in December 2014. Table 4 shows the mean values for actinidin contents in the ‘Hort16A’ and ‘Hayward’ kiwifruit.

SmartPill™ technology provides a measurement of pH; the temperature and pressure changes throughout gastrointestinal transport and the time for each period of gut transit are determined by changes in pH. 

**Figure 1:**
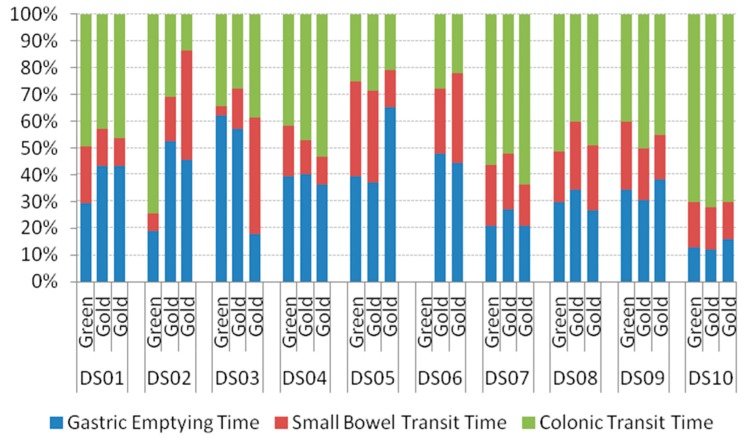
Gastric transit times (gastric emptying time, small bowel transit time, and colonic transit time) as a percentage of whole gut transit time. Each group of three (egDS01) represent an individual.

The gut transit times for each individual were analysed. The stacked bar chart (Figure 1) of the gastric emptying time, small bowel transit time, and colonic transit time (as a percentage of the whole gut transit time) demonstrates the large amount of inter-individual variability; there was no clear difference in transit times between the ‘Hayward’ and ‘Hort16A’ treatments.

Further analyses of variance on the transit times, blocked by participant and treatment order, confirmed the absence of differences between each treatment (Table 5).

VAS were used to assess subjective feelings of hunger and satiety over the course of the 6-h clinic visit: arrival, immediately after eating, and 60, 120, 180, 240, and 300 min after eating. The results for each individual were recorded and areas under the curve (AUC) for each participant-meal combination were calculated. There was no significant difference in the mean AUC between the ‘Hort16A’ and ‘Hayward’ kiwifruit (Table 6).

Additionally, the subjective rating of each feeling over the time of the trial visit was analysed. As expected, hunger and prospective food consumption decreased and fullness and satisfaction increased immediately following the consumption of the test meal. There was, however, no significant difference in the mean ratings of hunger, fullness, satisfaction, or prospective food consumption between the ‘Hort16A’ and the ‘Hayward’ treatments when measured over the 5 h between the test meal and the ad libitum lunch.

The mean total energy intake (EI) and the energy contributed by carbohydrate (CHO), fat, and protein, respectively, at the ad libitum lunch are presented in Table 7. There was no significant difference in total energy intake, CHO, fat, or protein between the ‘Hayward’ and ‘Hort16A’ kiwifruit treatments (*p* > 0.05).

At each visit, the participants were asked about five measures of gastric discomfort (pain in upper abdomen, rumbling in stomach, bloating, belching, and flatulence), using a seven category rating scale (from ‘no discomfort’ through ‘minor discomfort’, ‘mild discomfort’, ’moderate discomfort’, and ‘moderately severe discomfort’ to ‘severe discomfort’ and ‘very severe discomfort’; these were recorded as 0–6). These questions were asked on arrival, immediately after eating, and 60, 120, 180, 240, and 300 min after eating. Results for each individual were recorded and AUCs for each participant-meal combination were analysed (Table 8). Bloating was significantly reduced following the green kiwifruit.

Participants reported more bloating after the meals with ‘Hort16A’ kiwifruit than with ‘Hayward’ kiwifruit (*p* ≤ 0.05). This higher incidence of bloating was consistent with further analysis of the data. Using a binomial generalised linear model, the data were analysed to investigate the number of times at which participants simply reported any discomfort (Table 9). Again, there was more bloating reported following the consumption of the ‘Hort16A’ kiwifruit than the ‘Hayward’.

## 4. Discussion

This study examined the acute effect of kiwifruit (‘Hayward’ versus ‘Hort16A’) on digestion in healthy males, using SmartPill™ technology and subjective ratings of satiety and gastric comfort. Ten healthy males completed the study. The consumption of the ‘Hayward’ and ‘Hort16A’ kiwifruit resulted in no significant difference in gastric emptying time, small bowel transit time, or colonic transit time in this study (*p* > 0.05). Similarly, no significant difference was observed in visual analogue scale satiety measures (hunger, fullness, satisfaction, desire to eat, and amount they desired to eat), which was supported by the lack of significant difference (*p* > 0.05) in energy consumption at the subsequent ad libitum meal. There was an indication that the sensation of bloating may be reduced following the consumption of the steak meal with ‘Hayward’ kiwifruit (*p* < 0.028). This is consistent with research that has shown that ‘Hayward’ kiwifruit is effective in enhancing protein digestion both in vivo and in vitro because of the presence of actinidin. This activity helps to promote the physiologically active nature of kiwifruit in the gastrointestinal tract, potentially leading to enhanced digestion and an increase in digestive comfort [2,3]. The results of this study warrant further investigation to understand the effect of kiwifruit on bloating and other measures of gastric comfort with a larger sample size. This study was restricted to a small number (*n* = 10) because of the inclusion of SmartPill™ technology, which requires specialist equipment and is an expensive diagnostic tool.

This current study was a pilot study. SmartPill™ uses a non-digestible wireless transmitting capsule, with a receiver acquiring and storing signals from the capsule and software that displays the data on a computer. The capsule samples and transmits pH, pressure, and temperature data at regular intervals to the receiver worn by the participant. This is usually for a period of five days, after which the capsule is passed painlessly and naturally. It is generally used as a diagnostic aid for patients presenting with gastrointestinal disorders such as functional constipation to determine if and where gastrointestinal motility is affected. Currently there are no other reported studies using this technology in clinical studies for the purpose of assessing gastric transit times following a high protein (64%) meat meal, and thus the sensitivity of this technology for use in this manner was not guaranteed. The technology has been used in studies using a standard meal consisting of scrambled egg, bread, and jams with a protein content of 24% and demonstrated a good correlation with scintigraphy, the standard method for evaluating gastric function [12]. Key differences between that study by Kuo et al. and this current study were that, in the Kuo at al. study, the SmartPill™ capsule was swallowed prior to consumption of the meal and the second meal after 6 h was a liquid formulation specifically used to disrupt the emptying of the original test meal.

Timms et al. (2011) [13] conducted a controlled crossover trial to determine whether the SmartPill™ device could detect a significant difference in transit time when ten healthy subjects consumed 9 g of wheat bran or an equal volume of low-fibre control for three days. Colonic transit time decreased by 10.8 h (*p* = 0.006) with the wheat bran treatment. Whole-gut transit time also decreased by 8.9 h (*p* = 0.02) after the consumption of the wheat bran treatment. Gastric emptying time and small-bowel transit time did not differ between treatments. The results from the study by Timms et al. differs from the current study in that there was no difference in any of the colonic transit times, whole gut transit times, gastric emptying times, or small bowel transit times between the ‘Hort 16A’ and ‘Hayward kiwifruit’ meals. It could be that the difference between the wheat bran and low fibre control diets used by Timms et al. was greater than the differences between the ‘Hort 16A’ and ‘Hayward kiwifruit’ meals. Additionally, Timms et al. fed the participants the wheat bran and low fibre control for three days, whereas the current study only examined one meal, which may not have been a long enough time period to detect differences in transit times using the Smartpill™.

Normal gastric emptying rates are under 4 h in healthy subjects; however, this current study showed that there was a significant delay in gastric emptying rates across all treatments, with the mean time for ‘Hayward’ kiwifruit being 12 h and 14 h for the ‘Hort16A’ treatment. This suggests that the SmartPill™ capsule itself was being affected by the consistency of the meal or that the consumption of subsequent meals disrupted the emptying of the capsule. The steak was presented to participants in a bowl containing 400 g of meat in 1.5 cm cubes. Thus the steak volume was large and had a bulky texture, which may have physically prevented normal passage of the capsule through the stomach to the duodenum. This was then further compounded by the consumption of the ad libitum lunch 6 h after the capsule ingestion, translating into a further delay of transit times and high variability. Studies have previously shown that gastric emptying patterns are widely affected by the physical properties of foods, with minced beef being shown to digest more rapidly than beef steak [14,15]. Thus the format and volume of the meat meal was likely to have been a major limiting factor in demonstrating gastric emptying with this technology. 

Gastric digestion is a complex process involving both physical and chemical food breakdown. Gastric acid softens food particle texture, and digestive enzymes begin the hydrolysis of nutrients so they can be absorbed once the food reaches the small intestine [16]. Changes in pH are the only means of determining when the Smart Pill™ has left the stomach and arrived in the duodenum. At this point in time, there is a dramatic change in pH from a level as low as 2–3 to a neutral pH. The changes seen in the Smart Pill™ tracings are compelling, and this has led to the Smart Pill™ now being used to evaluate not only gastric emptying but whole gut motility by the use of pH as a continuous measurement.

In summary, there was no significant difference in gastric emptying times following a steak meal with either ‘Hayward’ or ‘Hort16A’ kiwifruit, as measured using SmartPill™ technology. Other technologies such as scintigraphy may be more appropriate to determine the effects of kiwifruit on rates of protein digestion and gastric emptying. Subjective ratings of satiety (hunger, fullness, satisfaction, desire to eat, and the amount they desired to eat) showed no significant difference between the two groups of participants, following the meal with either ‘Hayward’ or ‘Hort16A’ kiwifruit, which was supported by a lack of significant difference in the subsequent ad libitum meal. There was some evidence to suggest that the subjects reported feeling less bloated following the meal when it was consumed with ‘Hayward’ kiwifruit, which warrants further investigation.

## Figures and Tables

**Table 1:** Content of the standard meal eaten before each study day.

Food Description	Energy Content (kJ)
Watties Soup for One (tomato flavour)	810
Bread roll (wholemeal)	778
Watties Frozen Cottage Pie (400 g)	1440
Viguer Dairy Food (chocolate flavour)	597
Go Fruity peaches in natural juice	185
Nice ‘n’ Natural Muesli bar	818
Water (750 mL)	0

**Table 2:** Energy content and macronutrient content of foods offered to participants at the ad libitum lunch meal.

Food	Portion Size	Typical No. of Serves	Energy kJ	Protein (g)	Fat (g)	Carbohydrate (g)
Uncle Ben’s individual fried rice	500 g	2	4125	20	16.88	184
Bread Roll, white (Bun)	4 buns	4	3852	29.52	10.8	169.2
Bread Roll, mixed grain (Long Roll)	4 buns	4	3785	36	10.3	154.3
Chicken breast, deli-cooked	200 g	5	1100	36	10	4.8
Ham sliced, premium	200 g	5	902	33	6	7
Cucumber, raw	80 g	1	37	0.5	0.08	2
Tomato	100 g	1	82	0.75	0.4	4
Lettuce, inner leaves	10 g	1	2.5	0.06	0.2	0.09
Peaches in juice, small pottles	425 g	4	742	3.4	0	50.5
Yoghurt, vanilla bean flavoured	300 g	2	1116	12.8	6.1	45
Cookie Time original	100 g	4	2268	8.82	22.5	80
Butter (salted)	90 g	9	2747	0.41	74	0.7
Dressing—mayonnaise	90 g	9	1213	0.6	25	16.7
Soy sauce	90 g	9	130	5.4	0	4.3
Juice, Apple & Orange	1000 mL	10	1920	2	0.21	117
Water	1500 mL	10	0	0	0	0
**TOTAL**			17,897	189	182	624

**Table 3:** Baseline demographics of participants.

Baseline Characteristic	
Age in years (mean ± SD)	34 ± 10
Age (range)	21–48
Weight in kg (mean ± SD)	83 ± 12
Weight (range)	67–106
Body Mass Index (BMI) in kg/m^2^ (mean ± SD)	24 ± 3
BMI (range)	21–29

**Table 4:** Mean actinidin amount ^1^ in kiwifruit.

Batch Number	*Actinidia chinensis* var. *chinensis* ‘Hort16A’ (Marketed as Zespri^®^ Gold Kiwifruit)	*chinensis* var. *deliciosa* ‘Hayward’ (Marketed as Zespri^®^ Green Kiwifruit)
1 (November)	0.53 (0.031)	3.84 (0.13)
2 (December)	0.48 (0.039)	5.52 (0.18)

^1^ Showing µg azocasein digested per hour at 37 °C per mg kiwifruit homogenate; Mean (SD).

**Table 5:** Effect of intervention on each stage of gastrointestinal transit (hours).

Intervention	Gastric Emptying (h)	Small Bowel Transit Time (h)	Colonic Transit Time (h)	Small/Large Bowel Transit Time (h)	Whole Gut Transit Time (h)
*Actinidia chinensis* var. *chinensis* ‘Hort16A’	14	7.4	16.2	23.6	37.7
*A. chinensis* var. *deliciosa* ‘Hayward’	12.4	7.1	20.4	27.5	39.9
LSD	3.5	2.9	7.0	6.6	7.2
90% confidence interval	−1.3 to +4.5	−2.1 to + 2.7	−9.9 to +1.6	−9.3 to +1.6	−8.2 to +3.7
Intervention (1 *df*)	*p* = 0.490	*p* = 0.887	*p* = 0.439	*p* = 0.451	*p* = 0.722

LSD = Least squared difference between the two means at the 5% level; *df* = degrees of freedom.

**Table 6:** Mean area under the curve (AUC, cm.min) combined over the three meals.

Intervention	Hunger	Fullness	Satisfaction	Desire to Eat	Amount to Eat
*Actinidia chinensis* var. *chinensis* ‘Hort16A’	1109	1631	1726	1210	1211
*A. chinensis* var. *deliciosa* ‘Hayward’	1063	1643	1818	1126	1237
LSD (0.05)	287	289	230	259	258
90% confidence interval	−191 to 283	−251 to 227	−282 to 98	−130 to 298	−239 to 187
Intervention (1 *df*)	*p* = 0.739	*p* = 0.931	*p* = 0.411	*p* = 0.504	*p* = 0.829

LSD = Least significant difference between the two means at the 5% level; *df* = degrees of freedom.

**Table 7:** Mean intake over the three meals.

Intervention	Total Energy (MJ)	Carbohydrate (g)	Fat (g)	Protein (g)
*Actinidia chinensis* var. *chinensis* ‘Hort16A’	4.97	189	31	42
*A. chinensis* var. *deliciosa* ‘Hayward’	4.92	187	35	36
LSD (0.05)	0.68	29	7	8
90% confidence interval	−0.51 to 0.62	−22 to 26	−10 to 2	−1 to 10
Intervention (1 *df*)	*p* = 0.875	*p* = 0.871	*p* = 0.248	*p* = 0.110

LSD = Least significant difference between the two means at the 5% level; *df* = degrees of freedom

**Table 8:** Mean area under the curve (AUC) (scale points.minutes) over the three meals.

Intervention	Pain in the Upper Abdomen	Rumbling in the Stomach	Bloating	Belching	Flatulence
*Actinidia chinensis* var. *chinensis* ‘Hort16A’	68	77	150	80	85
*A. deliciosa chinensis* var. ‘Hayward’	56	50	84	77	82
LSD (0.05)	45	57	43	70	33
Intervention (1 *df*)	*p* = 0.559	*p* = 0.327	*p* = 0.005 *	*p* = 0.925	*p* = 0.811
On arrival rating (1 *df*)	*p* = 0.723	*p* = 0.154	*p* = 0.010 *	*p* = 0.902	*p* = 0.041*

LSD = Least significant difference between the two means at the 5% level; *df* = degrees of freedom; * significance at the 5% level.

**Table 9:** Mean values of participants’ reported discomfort.

Intervention	Pain in the Upper Abdomen	Rumbling in the Stomach	Bloating	Belching	Flatulence
*Actinidia chinensis* var. *chinensis* ‘Hort16A’	1.4	1.1	2.5	1.5	1.4
*A. chinensis* var. *deliciosa* ‘Hayward’	1.0	0.9	1.5	1.5	1.3
LSD (0.05)	0.7	0.6	0.9	1.0	0.7
Intervention (1 *df*)	*p* = 0.118	*p* = 0.452	*p* = 0.028 *	*p* = 0.926	*p* = 0.740

LSD = Least significant difference between the two means at the 5% level; *df* = degrees of freedom; * significance at the 5% level.
